# Improving medication safety for intensive care patients transitioning to a hospital ward: development of a theory-informed intervention package

**DOI:** 10.1186/s12913-024-11627-3

**Published:** 2024-11-26

**Authors:** Richard S. Bourne, Mark Jeffries, Jennifer K. Jennings, Darren M. Ashcroft, Paul Norman

**Affiliations:** 1 Departments of Pharmacy and Critical Care, Sheffield Teaching Hospitals NHS Foundation Trust, Herries Road, Sheffield, UK; 2 Division of Pharmacy & Optometry, School of Health Sciences, Faculty of Biology, Medicine and Health, The University of Manchester, Oxford Road, Manchester, UK; 3 National Institute for Health and Care Research (NIHR) Greater Manchester Patient Safety Research Collaboration (PSRC), School of Health Sciences, Faculty of Biology, Medicine and Health, University of Manchester, Oxford Road, Manchester, UK; 4 Department of Psychology, University of Sheffield, Portobello, Sheffield, UK

**Keywords:** Critical care, Medication safety, Transfers in care

## Abstract

**Background:**

Care of critically ill patients is complex, requiring effective collaboration co-ordination and communication across care teams and professions. Medicines are a fundamental component of the acute interventions intensive care unit (ICU) patients receive, requiring frequent review and optimisation according to patient needs. ICU patients recovering to transfer to a hospital ward are at risk of medication transition errors, contributing to poorer patient and health-system outcomes. We aimed to develop of a theory-informed intervention package to improve medication safety for ICU patients transferring to a hospital ward.

**Methods:**

We conducted a qualitative study comprising two UK face-to-face focus group meetings in April and May 2022. There were ten participants in each meeting (7-8 healthcare professionals and 2-3 patient and public representatives). Each meeting had four foci: (i) What needs to change (intervention targets)? (ii) What are the core intervention components? (iii) What will the intervention components change and how (mechanisms of action), and what key outcomes will the changes impact on? (iv) What are the barriers and facilitators to intervention delivery? A background to the problem and previous intervention development work was provided. Meetings were digitally recorded and transcribed verbatim. Iterative analyses, informed by the Behaviour Change Wheel framework, were conducted to provide a behavioural diagnosis, identify key behaviour change techniques and outline the mechanisms of action through which the intervention might impact on key outcome.

**Results:**

We identified what needs to change to improve medication safety for UK ICU patients on this care transition. A theory-informed intervention package was developed, based on seven core intervention components (e.g., medication review (targeted), task organisation and prioritisation). For each intervention component the mechanism of action, targeted change, and key outcomes were identified (e.g., medication review (targeted); action planning; decreases problematic polypharmacy; decreased preventable adverse drug events). Barriers and facilitators to intervention component delivery were described.

**Conclusions:**

We developed a theory-informed core intervention package to address the limitations in medication safety for ICU patients transferring to a hospital ward. Understanding what needs to change, and the accompanying facilitators provides a basis for intervention feasibility testing and refinement prior to future evaluation of effectiveness.

**Supplementary Information:**

The online version contains supplementary material available at 10.1186/s12913-024-11627-3.

## Background

Care of critically ill patients in intensive care units (ICUs) requires effective collaboration, co-ordination and communication across multiple healthcare teams and professions. Multi-organ support for patients involves responsive and repeated adjustments to interventions including organ support systems and medication. For patients surviving intensive care, the recovery pathway can be challenging, being prone to adverse events [1], and poor long-term outcomes [2, 3]. As such, unplanned readmissions to hospital after discharge are common [4, 5], adding to the healthcare demands for survivors [3]. Long-term outcomes are impacted by a range of acute and chronic factors [6], with multi-morbidity [2, 6] and polypharmacy [4] risk factors for unplanned hospital readmissions [5].

Medication is the most common intervention ICU patients receive, requiring multiple changes to acute and long-term chronic medication during their care episode. The extent of these medication changes predisposes patients recovering from a critical illness to medication transfer errors leading to problematic polypharmacy comprising of inappropriate continuation of acute medication no longer indicated and failure to restart important chronic medications [7–10]. These medication transfer errors can contribute to poorer outcomes for ICU survivors such as early unplanned hospital readmission [11, 12].

Several interventions have been evaluated to improve medication safety for ICU patients transferring to a hospital ward [13]. Multicomponent interventions based on staff education and guidelines reduce the risk of inappropriate continuation of acute medication on hospital discharge [13]. Such interventions may be easier to implement but are not designed to address the perceived greater clinical risk, that is, failure to re-introduce and optimise medication for pre-existing illnesses after a critical care episode [14]. Compared to other hospitalised patients, critical care patients are at increased risk of discontinuation of important pre-existing medication such as cardiovascular medicines [15]. More complex interventions are required to meet the less predictable medication needs of patients recovering after a critical illness in the context of pre-existing multimorbidity, along a recovery pathway provided by multiple care teams [16–18].

Development of a complex intervention should be based on identifying the research evidence and underpinning with theory, including processes and outcomes [19, 20]. Our previous work on medication safety for ICU patients transferring to a hospital ward highlighted several medication and systems-related risks, including limitations in delivery of medication reviews, effective communication of plans and care continuity [13, 14, 16, 21]. In the current study, we build on this work by identifying the core intervention priorities to develop an intervention package to improve medication safety in recovering ICU patients on hospital ward transfer [13, 14, 16, 21]. In particular, we aimed to (i) identify what changes in systems and processes were required, (ii) confirm the core medication-related intervention components for use in UK clinical practice, (iii) understand the theory of how the mechanisms through which the intervention components may lead to targeted changes and key outcomes and, (iv) identify what are the barriers and facilitators to delivering the intervention components in routine clinical practice.

## Methods

### Focus group meetings and participants

We conducted a qualitative study comprising two focus group meetings in April and May 2022 in Sheffield and London, respectively. Ten UK participants (7 to 8 healthcare staff and 2 to 3 patient and public) provided written informed consent prior to attending each focus group meeting. Purposive sampling of participants was used. Participants were primarily recruited by email by RSB from panels that had completed an earlier international Delphi consensus process on important medication safety intervention components and outcome measure for ICU patients transferring to a hospital ward [21]. A minority of participants were recruited via advertisements to ensure availability of diverse participant attendance at the focus group meetings. Professionally diverse UK ICU and hospital ward healthcare staff involved in medication review and prescribing activities were represented alongside public representatives with lived experience of emergency and critical care (Table 1). These healthcare professional groups represented the core active agents delivering medication safety interventions on transition from ICU to the hospital ward, according to local context and staff workforce [16, 21]. Both focus group meetings were held face to face in central city locations for ease of participant travel, and each meeting was conducted over four hours with encrypted digital recording. Each focus group meeting was led by RSB (clinical-academic pharmacist specialising in critical care), facilitated by the wider research team (MJ (qualitative researcher specialising in medication safety), JKJ (critical care pharmacist) and PN (health psychologist specialising in behaviour change)). The meetings included a background to the medication safety challenges for ICU patients transferring to a hospital ward, a summary of our research findings to date [13, 14, 16, 21], the meeting aims and objectives, then a brief overview of behaviour change science. Participant ground rules were explained including equal value of contributions and facilitators encouraged contributions from all participants. Topic guides were developed in advance of each focus group meeting based on discussion within the research team of the findings from our previous studies [13, 14, 16, 21], to ensure research objectives were met [Additional file]. There were four sections to the meeting discussions: (i) What needs to change (intervention targets)? (ii) What are the core intervention components? (iii) What will the intervention components change and how (mechanisms of action), and what key outcomes will the changes impact on? (iv) What are the barriers and facilitators to intervention delivery? In the Sheffield meeting, we used tasks such as *1–2-4-All* [22] to engage and help participants reach consensus on the core intervention components [Additional file]. The findings from the Sheffield meeting were shared and further discussed at the London meeting to ensure confirmation of results. The consolidated criteria for reporting qualitative research (COREQ) reporting guidelines were used [23].

**Table 1:** Focus group participants by meeting location

**Participants**	**Sheffield focus group**	**London focus group**
**ICU Pharmacist**	2	3
**Ward Pharmacist**	2	-
**ICU Doctor**	-	1
**Ward Doctor**	-	1
**Advanced Critical Care Practitioner**	2	-
**Outreach Team Member**^a^	1	2
**Patient and public representative**	3	2
**ICU Nurse**	-	1

^a^Outreach Team Members were critical care trained nurses or advanced nurse practitioners working within a critical care outreach team

### Data confirmation and analysis

The discussion foci (i-iv) of the meeting recordings were transcribed verbatim. The Behaviour Change Wheel [24], provided the overarching theoretical framework for the analysis to aid the behavioural diagnosis (i.e., what needs to change), identify intervention options and content (i.e., where and how to intervene), and outline the mechanisms through which the intervention might impact on key outcomes. Analysis involved immersion in the data, identification of content, review and discussion across the research team. Transcripts from both meetings were read independently by two researchers (JKJ and MJ) to identify the content of the findings for each section. Next, the research team (RSB, JKJ, MJ) met to review the identified findings from the transcripts and confirm the content. The findings were then further discussed across the research team (RSB, JKJ & MJ) specifically to finalise the targeted changes and outcomes. This was followed by identification of the specific mechanisms of action of the intervention components and linkage to targeted changes from the transcripts (RSB, MJ). This process drew upon the Behaviour Change Wheel framework [24]. Using an iterative process, the graphical presentation of the intervention components, mechanism of action, targeted changes and key outcomes were discussed and refined across the research team in several meetings [Additional File; Figure A1 and Table A1]. Finally, the barriers and facilitators to implementation of each intervention components were categorised using a patient safety systems framework (Systems Engineering Initiative for Patient Safety, model 3.0) to support understanding and actioning [25]. These were discussed and agreed by the research team before dissemination to all focus group participants to ensure agreement. Participants confirmed their agreement with the findings as presented and no changes were requested.

## Results

### What needs to change

Participants identified several areas and components that required change to improve medication safety for ICU patients transferring to a hospital ward (Table 2). Areas that required improvement were communication and collaboration, medication planning, knowledge and skills, tools and technologies. Increased clarity and understanding were required in staff roles and responsibilities in medication safety on this interface in patient care. Linked to this was the importance of staff understanding potential consequences (patient and organisational) if no system or process changes were made. The system needed to be more proactive around medication review and planning, including in prioritisation decisions, whilst managing and anticipating predictable time pressures. The need for increased engagement with the patient and family around medication changes and hospital ward transfer was also identified.

**Table 2:** What needs to change in current UK ICU and hospital practice for transfer medication safety

**Communication and collaboration (improve)**	Dynamic communication within ICU care team and between care teams
	Handover to hospital ward staff (with appropriate knowledge and skills)
	Structured communication and recommendation (action plan)
	Accessibility of information, with written documentation complimenting verbal communication
**Medication planning (improve)**	Formulation of medication plan by ICU care team, effectively communicated with appropriate hospital ward staff
	Access to healthcare professionals with appropriate knowledge and skills to support decision making around medicines
**Roles and responsibilities (increased clarity and understanding)**	Healthcare professionals aware of their own roles and responsibilities and those of others (delegation and ownership) in medication safety
**Knowledge and skills (improve)**	Healthcare professionals’ awareness and understanding of importance of medication safety and continuity for patients on transfer from ICU
	Availability of appropriate healthcare professionals with ability to support decision making around medicines use
	Accessibility of relevant information
	Education and training of healthcare professionals around medication safety on interfaces of care
	Importance of permanent (non-rotational) healthcare professionals as trainers and delivery of service continuity
**Tools and technologies (improve)**	Support medication-related tasks and processes (e.g., automation)
**System (more proactive)**	Medication review component of routine daily practice of healthcare professionals and multiprofessional ward rounds
	Appreciate the complexity of ICU patients and their medicines on transfers in care
	Collaboration across and between care teams with appropriate specialist referral as needed (e.g., acute pain team, critical care outreach team)
**Time pressures (manage, anticipate)**	Medication review in routine practice improves preparation and planning for patient transfer
	Acknowledge high workload and healthcare professional staffing pressures
	Daytime patient transfers preferred
**Prioritisation (of medication review)**	Medication review part of routine daily working practice
	Understanding role of medication review in overall patient care needs
	Designated healthcare professional groups lead on medication review based on knowledge and skills
**Patient and family engagement (increase)**	Timing important, based on patient care trajectory and preparation for transfer to a hospital ward
	Must be individualised according to patient acuity of illness and capability to participate
	Family members engaged when appropriate to support the patient
	Patient/ family informed of medication plan and encouraged participation as part of the patient’s recovery
**Potential consequences if no changes (understand)**	Learning from preventable medication errors
	Audit and feedback system (including patient stories)

### Core intervention components

Participants were able to prioritise the core intervention components derived from important medication safety interventions identified in a previous Delphi consensus process [21]. The intervention package comprised of seven intervention components and related ingredients (Table 3). The intervention components being: Medication review (targeted), education and training, communication and collaboration (around medicines), guidelines (medication transfer), task organisation (and prioritisation), team organisation and documentation of the medication plan.

**Table 3:** Intervention package composed of the core intervention components

**Medication review (targeted)**	**Education and training**	**Guidelines (medication transfer)**	**Team organisation**	**Task organisation (and prioritisation)**	**Communication and collaboration (around medicines)**	**Documentation of medication plan**
Medicines reconciliation on admission to and transfer from ICU Daily medication review whilst on ICU Medication review prior to transfer from ICU Medication review on admission to hospital ward	Education of both ICU **and** ward staff on • Medication at high-risk for inappropriate continuation on transfer to a hospital ward • Medication at high-risk for failure to restart on transfer to a hospital ward	Guidelines (ICU/ hospital) on short-term ICU medication, including indication and when to stop or wean off ICU to hospital ward transfer protocol with medication section	Daily multi-professional ward rounds on ICU with attendance by all appropriate healthcare professions involved in patient medication review All ICU clinical staff are aware of i) their own roles and responsibilities, and ii) the roles and responsibilities of other healthcare professionals, in the safety and continuity of medication on ICU transfer	All appropriate ICU staff aware of decision that the patient is ready for transfer from ICU to a hospital ward Appropriate delegation of medication review and transfer tasks (related to staff knowledge and skills) Keep patient/ family updated/ informed of what is happening/ changing	Verbal structured handover of medication therapy by: • ICU medical team to hospital ward team • ICU nursing team to hospital ward team • Verbal or electronic handover of medication therapy information and review requirements by ICU pharmacy team to ward pharmacy team Refer to specialist teams (if required)	Mandatory medication checklist Medication transfer summary report Documentation of: • Antimicrobial(s) indication(s), start dates and review date(s) included • Medication permanently discontinued and reasons • Medication intended to continue • Medication (chronic/ long-term), documentation of criteria to restart/re-titrate • Medication temporarily held/omitted and reasons • Medication dose and route changes and reasons

### Inter-relationship between intervention components, targeted changes, and key outcomes

**Fig. 1:**
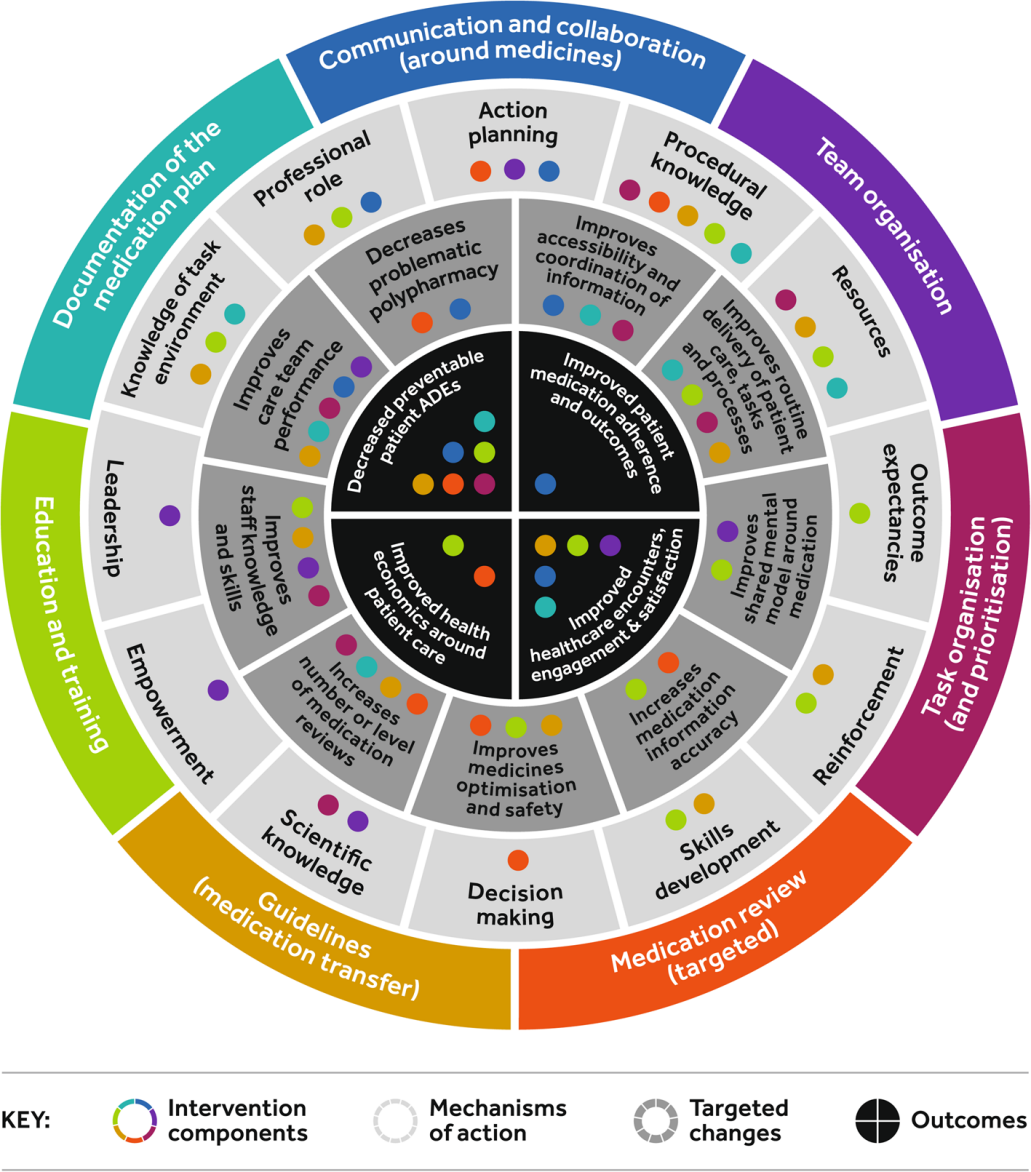
Relationship between the intervention components, mechanism of action, targeted changes, and outcomes by intervention component

**Fig. 2:**
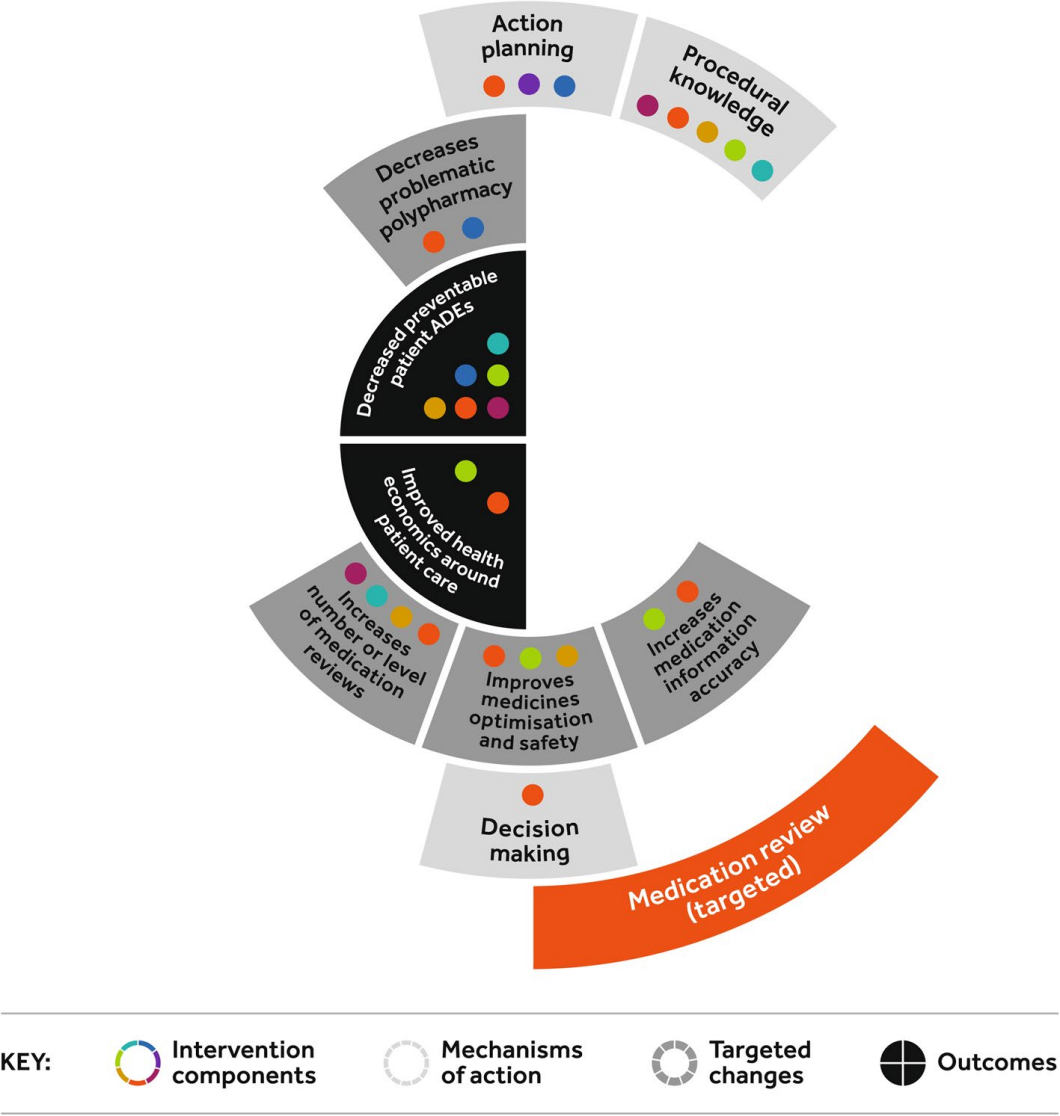
Relationship between the *Medication Review* intervention component, mechanism of action, targeted changes, and outcomes

For each intervention component of the intervention package, participants identified the mechanisms of action [24], through which the component would impact on the targeted changes and, in turn, key outcomes (Figs. 1, 2 and Figures A2-8 Additional file). Figure 1 represents the complexity of the medication safety intervention package and inter-relationships leading to the targeted changes and then key outcomes. The relationships for each individual intervention component are represented in Figures A2-8 [Additional file]. *Procedural knowledge* was the most common mechanism of action followed by *resources*. *Action planning*, *professional role* and *knowledge of task environment* were identified as important behaviour change techniques that could be used to promote change in the mechanisms of action across different intervention components and support implementation. The four final outcomes captured the benefits for patient care (*decreased preventable adverse drug events; improved medication adherence and outcomes; improved healthcare encounters, engagement and satisfaction*), and for the health care system (*improved health economics around patient care* provided e.g., reduced unplanned hospital readmissions). The most common positive anticipated outcomes from the intervention package were*, decreased patient ADEs and improved healthcare encounters, engagement and satisfaction*. In particular, the proposed impact of improved *team organisation*, through *action planning, empowerment, leadership and scientific knowledge*, targeting *improves shared mental models around medicines, staff knowledge and skills and care team performance*; contributing to *improved healthcare encounters, engagement and satisfaction* for healthcare professionals and patients. As such, team organisation seemed a fundamental component to the successful delivery of the overall intervention package. Similarly, the most frequent targeted change the intervention package impacted on was *improves care team performance*, underlining the importance of team organisation and performance in the delivery of improved medication safety. The Medication Review (targeted) intervention component relationships are presented as an example (Fig. 2).

### Barriers and facilitators to intervention component delivery

The barriers and facilitators identified for each intervention component and patient safety systems categorisation are shown in Table 4. Examination of the barriers and facilitators shows a high degree of overlap with what needs to change (Table 2) to improve medication safety on this interface of patient care. Most barriers and facilitators to delivery of the intervention package related to the care team, organisational conditions and tools and technologies.

**Table 4:** Barriers and facilitators to routine delivery of the intervention components in routine UK clinical practice categorised by patient safety systems

**Intervention Component**	**Barrier**	**Facilitator**
**Medication Review (targeted)**	**Care Team** Lack of understanding of clinical importance Limited knowledge of medicines amongst some healthcare professional groups **Organisational Conditions** Lack of time **Tasks** Lack of healthcare professionals capable of making decisions around medicines **Tools and Technologies** Lack of ease of access to contemporary information Limited e-prescribing functionality	**Care Team** Knowledge and skills around medicines and related processes **Organisational Conditions** Multiprofessional teams Specialist teams, including outreach team **External Environment** National guidelines e.g., NICE NG5 **Tasks** Clearly documented medication plan
**Education and training**	**Care Team** Lack of appreciation of clinical importance Limitations in knowledge around non-specialist medicines **Tools and Technologies** Audit and feedback **Organisational Conditions** Lack of time Non-permanent (rotating) healthcare professionals Demands of other organisational training priorities Intensity and complexity of training programme	**Tools and Technologies** Patient stories Audit and feedback **Organisational Conditions** Education and training programme with competency check Permanent healthcare professional staffing models
**Guidelines (medication transfer)**	**Organisational Conditions** Too many guidelines	**Tools and Technologies** Accessible and easily readable **Organisational Conditions** Imbedded into routine clinical practice Supported by education and training
**Team organisation**	**Care Team** Low clinical priority of medicines in some healthcare professional teams **Organisational Conditions** Limitations in time/ high healthcare professional workload Healthcare professionals shift working Inadequate healthcare professionals staffing levels (e.g., clinical pharmacists)	**Care Team** Appropriate team task delegation and prioritisation Good collaboration between ICU and hospital ward/ specialist teams **Organisational Conditions** Structured day Permanent (non-rotational) team—know their roles and responsibilities Access to specialist teams
**Task organisation (& prioritisation)**	**Care Team** Limitations in healthcare professional beliefs about consequences and subject knowledge **Tools and Technologies** Lack of integration and functionality IT systems (incl. e-prescribing) **Organisational Conditions** Opportunity costs Challenges to maintaining a skilled workforce Out of hours patients transfers Poor transfer planning	**Care Team** Increased staff beliefs about consequences and subject knowledge Healthcare professionals’ knowledge of high-risk patients Positive team culture **External Environment** National service specification encouraging daytime transfers National critical care healthcare professional staffing recommendations e.g., Outreach Teams Link to demonstration of training programme competency **Organisational Conditions** Standardisation of practice Patient and family engagement and ownership **Tasks** All healthcare professionals aware of patients transfer plans **Tools and Technologies** Audit and feedback Medication transfer checklist
**Communication and collaboration (around medicines)**	**Care Team** Healthcare professional silo working **Tasks** Lack of documentation Excessive complex information for patient/family **Tools and Technologies** Lack of integration of IT systems	**Care Team** Within the whole multiprofessional ICU team **Tasks** All healthcare professionals aware of patient transfer plans Clear documentation of medication record Dynamic communication (collaboration) Appropriate use of plain English language with patients/ families **Tools and Technologies** Accessibility of information Standardised handover (Healthcare professionals know how and what to communicate; verbal and written modes) Technology assisted/ automated
**Documentation of medication plan**	**Organisational Conditions** Lack of time, especially for long-stay or complex patients **Tasks** Inadequate or ineffective communication	**Care Team** Healthcare professionals’ collaboration **Tasks** Prescription annotation **Tools and Technologies** Structured handover process

Examples of indicative quotes that informed the what needs to change, the core intervention components, mechanisms of actions and barriers and facilitators to the intervention component delivery are provided (Table 5).

**Table 5:** Examples of indicative quotes that informed the what needs to change, the core intervention components, mechanisms of actions and barriers and facilitators to the intervention component delivery

**Indicative quotes**	**Results development**
*“Standardisation: try to standardise the communication that is used throughout”* [R5 London] “*a good plan empowers*” [R6 London]	**WNTC:** Communication and collaboration (improve); Structured communication and recommendation (action plan) **IC:** Documentation of medication plan **MoA:** Action planning **B&F:** *Barriers* Tasks: Inadequate or ineffective communication *Facilitators* Care Team: Healthcare professionals’ collaboration Tools and Technologies: Structured handover process
*“anyone above an F2 gradespends all day in theatres because they’re training to be surgeons* […] *and most of the senior doctors from the surgery don’t know how to use that prescribing system at all. So they don’t look at it. So it’s down to the F1s completely to do all this sort of stuff. And often if something is omitted on ITU because the patient’s not safe, for example, anti-hypertensives, they might come down to the ward with them omitted, and every three months when the doctors rotate I hear the same thing, oh, well, ITU have omitted it so it must be really important, and who am I to question ITU?”* [R3 Sheffield] “I feel that’s the problem; I think you can be really good at communicating, but if the people that you are communicating (to) don’t understand” [R3 London]	**WNTC:** Knowledge and skills (improve); Access to healthcare professionals with appropriate knowledge and skills to support decision making around medicines **IC:** Medication Review (targeted): Medication review on admission to hospital ward; Documentation of medication plan: Medication transfer summary report; Education and training: Education of both ICU and ward staff **MoA:** Procedural knowledge **B&F:** *Barriers* Care Team: Lack of understanding of clinical importance, Limited knowledge of medicines amongst some healthcare professional groups Tasks: Lack of healthcare professionals capable of making decisions around medicines *Facilitators* Care Team: Knowledge and skills around medicines and related processes Tasks: Clearly documented medication plan
*“it’s such complex information, isn’t it?….you communicate with one staff nurse at eight o’clock at night, by the time the following morning comes at the end of her night shift, her priorities are totally different, and also the time pressure is added at each point.”* [R4 Sheffield] “*every time I go to a new hospital there is one system for ICU, one or two different systems for the ward, depending on which ward you’re working on, that you have to learn a whole new system from scratch*” [R5 London]	**WNTC:** Structured communication and recommendation (action plan) **IC:** Documentation of medication plan: Medication transfer summary report **MoA:** Knowledge of task environment **B&F:** *Barriers* Tasks: Inadequate or ineffective communication *Facilitators* Care Team: Healthcare professionals’ collaboration Tasks: Prescription annotation
*“daytime is when all of the key stakeholders in the process are there [..] And what can happen is if the patient then disappears out of hours all this information is handed over, the follow-up then is expected to potentially be followed by a general surgical or SHO or something. And it’s just not very robust. But there’s sometimes a bit of apathy from the intensivist, well because there’s been an opinion that it’s done and it will be followed up”* [R6 London] *“it’s interesting about what time patients are being discharged and time of discharge and who you’re handing over to, because if they’re handing over to somebody within daytime hours and then your patient’s going out of hours, so that medic’s not going to see that patient when they get to the ward so what’s passed over to out of hours team…and if that medic on the next day to like pick up”* [R7 Sheffield] “I still think you’d be under time pressures because I don’t think you get the funding necessarily to operate a whole normal weekday service at the weekend, but seeing the high risk safety issue is really important” [R3 Sheffield]	**WNTC:** Time pressures (manage, anticipate) **IC:** Task organisation (and prioritisation): All appropriate ICU staff aware of decision that the patient is ready for transfer from ICU to a hospital ward **MoA:** Resources **B&F:** *Barriers* Care Team: Limitations in healthcare professional beliefs about consequences and subject knowledge Organisational Conditions: Out of hours patients transfers; Poor transfer planning *Facilitators* Care Team: Increased staff beliefs about consequences and subject knowledge. Healthcare professionals’ knowledge of high-risk patients. Positive team culture Tasks: All healthcare professionals aware of patients transfer plans
*“You need to strike a balance between informing the patient and listening to the patient and overburdening the patient at a difficult time.”* [R9 Sheffield] “*So when is the right time to engage about conversation about medicines and medicines changes? Because actually they might just be processing what’s… I’ve fallen into the trap as a junior pharmacist going well, I’ve got an agenda here to talk about medicines, but actually I think that person’s still processing what’s just happened to them and therefore is it about the timing of talking to people about that, and that may be very individual, but I think we need to be very sensitive to that, don’t we, that that would be different for different people*.” [R5 Sheffield]	**WNTC:** Patient and family engagement (increase) **IC:** Task organisation (and prioritisation): Keep patient/ family updated/ informed of what is happening/ changing **MoA:** Procedural knowledge; Resources **B&F:** *Barriers* Care Team: Limitations in healthcare professional beliefs about consequences and subject knowledge Organisational Conditions: Opportunity costs *Facilitators* Care Team: Increased staff beliefs about consequences and subject knowledge; Healthcare professionals’ knowledge of high-risk patients; Positive team culture Organisational Conditions: Standardisation of practice; Patient and family engagement and ownership
*“The junior doctors are left […] often what will happen is the patient will get to the point of discharge before […] something happens or a nurse specifically requests a doctor reviews the medication list for some reason, so usually one of two triggers will happen with anti-hypertensives. Either the patient’s blood pressure will become so high that they will trigger on our observation scoring system […] or it comes to the point of discharge when the doctor’s clicking the medicines over to the discharge summary and then they’ll see that their anti-hypertensives haven’t been given the entire admission and then they’ll just re-prescribe them all at maximum dose to have when they leave”* [R3 Sheffield]	**WNTC:** Medication planning (improve) Formulation of medication plan by ICU care team, effectively communicated with appropriate hospital ward staff; Access to healthcare professionals with appropriate knowledge and skills to support decision making around medicines **IC:** Medication Review (targeted): Medication review on admission to hospital ward **MoA:** Procedural knowledge **B&F:** *Barriers* Tools and Technologies: Lack of ease of access to contemporary information *Facilitators* Care Team: Knowledge and skills around medicines and related processes
*“Education….could be incorporated a little bit more, so where we learn actually from the errors that are taking place and then you’ve got more evidence based learning. You see that, oh that’s a high risk medication, that happened to that patient, let’s be sure that that’s actually what we mean. And I think education for ward based staff and staff in ICU that would be where learning from those areas would come from in terms of improving medication management”* [R9 London]	**WNTC:** Potential consequences if no changes (understand). Learning from preventable medication errors **IC:** Education and training: Education of both ICU and ward staff on medication at high-risk for inappropriate continuation on transfer to a hospital ward and medication at high-risk for failure to restart on transfer to a hospital ward **MoA:** Skills development; Outcome expectancies **B&F** *Barriers* Care Team: Lack of appreciation of clinical importance *Facilitators* Tools and Technologies: Patient stories
*“It’s not just a technical task; it is a real impact.” [R2 London]* *“It’s all encompassing, isn’t it, a meds (medicines) rec (reconciliation)”* [R1 Sheffield]	**WNTC:** Knowledge and skills (improve). Healthcare professionals’ awareness and understanding of importance of medication safety and continuity for patients on transfer from ICU **IC:** Medication Review (targeted): Medication review prior to transfer from ICU **MoA:** Decision making **B&F:** *Barriers* Care Team: Lack of understanding of clinical importance. Limited knowledge of medicines amongst some healthcare professional groups *Facilitators* Care Team: Knowledge and skills around medicines and related processes
*“some specialties are better at that than others. They look at their patients that are looking towards the end of ITU and well enough to come out. And they tend to be a vested interest. And then some specialties stand way back and just assume that ITU will have fixed it and discharged it.”* [R8 London]	**WNTC:** System (more proactive). Collaboration across and between care teams with appropriate specialist referral as needed (e.g., acute pain team, critical care outreach team) **IC:** Team organisation; Communication and collaboration (around medicines) **MoA:** Professional role **B&F:** *Barriers* Care Team: Low clinical priority of medicines in some healthcare professional teams *Facilitators* Care Team: Appropriate team task delegation and prioritisation Good collaboration between ICU and hospital ward/ specialist teams
*“I think we need to do a bit more about ensuring that the issue of discharge reconciliation is really important around the whole healthcare community. And at the moment I don’t think it’s well known….that, one in four patients being readmitted within 90 days. So, I think there’s something about actually getting that message really out there onto a wider community and, including critical care and outside, so that they realise that this is important.”* [R2 London]	**WNTC:** Knowledge and skills (improve). Education and training of healthcare professionals around medication safety on interfaces of care **IC:** Education and training: Education of both ICU and ward staff on medication at high-risk for inappropriate continuation on transfer to a hospital ward and medication at high-risk for failure to restart on transfer to a hospital ward **MoA:** Outcome expectancies **B&F:** *Barriers* Care Team: Lack of appreciation of clinical importance. Limitations in knowledge around non-specialist medicines Organisational Conditions: Lack of time. Non-permanent (rotating) healthcare professionals. Demands of other organisational training priorities *Facilitators* Tools and Technologies: Patient stories Organisational Conditions: Education and training programme with competency check. Permanent healthcare professional staffing models
*“having in an ideal world a standardised system that transfers between ICU and the ward, that translates between them.” [*R4 London]	**WNTC:** Tools and technologies (improve). Support medication-related tasks and processes (e.g., automation) **IC:** Documentation of medication plan Medication transfer summary report **MoA:** Resources **B&F** *Barriers* Tasks: Inadequate or ineffective communication *Facilitators* Tools and Technologies: Structured handover process Tasks: Prescription annotation

*B&F* Barriers and Facilitators, *IC* Intervention Component, *MoA* Mechanisms of Action, *WNTC* What needs to change

## Discussion

Through the focus groups of UK healthcare professionals and patient and public representatives and drawing on the Behaviour Change Wheel framework [24], we were able to identify the key healthcare system changes required to improve medication safety for ICU patients transferring to a hospital ward. Whilst some were specific to medication (e.g. medication review and planning, staff knowledge and skills), others centred around team performance in the provision of quality continuity of care, such as communication and collaboration, task prioritisation and clarity in staff roles and responsibilities. Also highlighted was the importance of patient and family engagement about their medication. We then identified the critical components of an intervention package designed to address the required changes, centred on improving team and task performance, communication-specific targeted medication reviews and documentation, and underpinned by staff education and guidelines. The mechanisms of action and relationships with targeted changes and key outcomes were identified for these critical intervention components. The potential benefits of decreased preventable patients adverse drug events and improved healthcare encounters, engagement and satisfaction were emphasised. Finally, the patient safety systems categorisation [25] of the barriers and facilitators emphasised the importance of organisational conditions, care team and tools and technologies within the healthcare context.

We have previously reported that a simple, and easy to implement multicomponent intervention based on education of staff and clinical guidelines, was effective in reducing the risk of potentially inappropriate medication at hospital discharge [13]. Although the use of guidelines and education and training of healthcare professionals is clearly important, the current intervention package goes beyond this to include an expanded range of intervention components (e.g., medication review (targeted), team organisation) with mechanisms of action (e.g., procedural knowledge, action planning) that focus on a range of targeted changes (e.g., decreases problematic polypharmacy, improves shared mental models around medication) that are likely to impact on a number of key outcomes in addition to inappropriate medication (e.g., decreased preventable patient adverse drug events, improved healthcare encounters, engagement and satisfaction). The intervention package also encompasses the deprescribing facilitators identified by ICU healthcare professionals in another qualitative study exploring acute antipsychotic use for various ICU-related indications [26]. However, we have previously identified that the higher clinical risk is the failure to restart clinically important long-term medication [14]. Re-introduction of important long-term medication such as cardiovascular medicines, requires repeated patient evaluation and progress review across a patient’s individual recovery pathway [6, 18]. Understandably, interventions to address such uncertainty require a more complex, system and team-based response such as provided by a package of transfer tools [27]. In a multicentre randomised controlled trial in high-risk hospitalised patients with sepsis, a multicomponent intervention including a post-discharge medicines optimisation component improved a composite 30-day mortality and hospital readmission outcomes [28]. Medicines optimisation was delivered through co-ordinated medication review and reconciliation. Our intervention package is built on delivery of medicines optimisation for *all* critically ill patients.

We found that most intervention components targeted care team performance. The team organisation intervention component is instrumental to the overall working of the intervention package, leading to improved staff healthcare encounters, engagement, and satisfaction. Improving staff engagement positively impacts on patient safety [29]. Team organisation also addresses several elements of what needs to change and supports intervention components delivery including task organisation (and prioritisation), communication and collaboration, medication review (targeted) and (team based) education and training. Although this complex intervention focuses on improving medication safety, the package is likely to have wider patient safety benefits by improving system and care team performance [30]. A team-based approach also addresses an overreliance on a single profession to deliver medication safety interventions [13, 14], increasing the potential effectiveness.

The focus group participants felt that medication review and education and training of healthcare professionals is likely to have outcome benefits for patients (reduced preventable ADEs), improved patient healthcare encounters and had wider health system benefits via improved health economic outcomes. There are conflicting systematic review findings of patient outcome benefits from in-hospital medication review by pharmacists [31, 32]. However, a recent scoping review of economic evaluations of critical care pharmacy services reported cost-benefits from medication review activities [33]. The intervention package highlights multiprofessional medication review opportunities on admission, then daily (including on the multiprofessional ward round), prior to transfer from critical care as well as the need to involve specialist teams when indicated. This co-ordinated, team approach to medication review is likely to maximise effectiveness on patient outcomes and health economic benefits.

The need for a structured handover on transfer from critical care is known [34]. However, healthcare professionals do not always know what medication-related information to include in a handover [16]. Our intervention package builds on previous work in this area [21, 35, 36], to identify the critical medication-related information required in summary reports for ICU patients transferring to the hospital ward.

The intervention package also highlights the importance of engaging with the patient and family about their medicines to improve outcomes related to healthcare encounters, engagement and satisfaction. To date, interventions evaluated to improve medication safety for ICU patients continuing their recovery pathway have not considered this aspect of patient safety [13]. We have recently reported that patients and family members want to be engaged about their medication during recovery from a critical illness and this requires a tailored approach from healthcare professionals [37].

In common with other complex clinical environments, implementation of changes in clinical practice in ICU has several challenges [38]. Successful implementation of complex interventions is dependent on system and staff behaviour changes [19]. Acknowledging this, we have been cognisant of the importance of understanding the current limitations in the system performance and barriers to implementation component delivery. Identification of these factors, combined with the theory-informed basis of the intervention package provides a solid platform for further refinement in clinical practice intervention prior to formal effectiveness evaluation. By elucidating the *what needs to change*, we provide a focus for improvement efforts that compliment implementation strategies. We had previously identified the limitation of the staff beliefs about consequences of (poor) medication safety on this transition in patient care [16]. Similarly, Jaworska et al. [26], reported the role beliefs about consequences has on clinicians’ motivation to use antipsychotics in ICU patients, contributing to increased risk of inappropriate continuation throughout the patient hospitalisation care episode. The theory-informed basis can form part of the staff behaviour changes required here and can be complemented with facilitators such as audit and feedback and patient stories.

We identified barriers and facilitator for each of the intervention components that will assist further refinement and implementation within clinical practice. These barriers and facilitators build on previous work specific to medication safety on transitions in patient and complement those already identified for quality in patient care [39]. They also highlight the potential limitations of individual intervention components and requirement for a package of intervention components to increase the likelihood of success in improving medication safety. Further examination of the facilitators to team organisation and task organisation indicates the criticality of these elements in team performance and successful intervention delivery. Many of these facilitators can be addressed by organisational changes to improve staff shared mental models, so staff know the goal-oriented treatment plans, (team and task-related) can prioritise, then complete tasks and have awareness of their roles and responsibilities and those of multiprofessional colleagues [30, 40]. Similarly, the current findings underline the importance of having an integrated, functional electronic health record systems with easily accessible documentation and automation to facilitate communication and medication review tasks [16]. Electronic transfer tools can improve the completeness and timing of ICU patient transfer communication including medication-related elements [35].

The focus groups had several strengths. Firstly, the development of the intervention package built on previous intervention identification and development work undertaken on medication safety for ICU patients transferring to a hospital ward [13, 14, 16, 21]. The focus group panels included the diverse and key ICU and ward-based healthcare professionals involved in medication review and prescribing for patients on this interface of care, as well as patient and public representatives, drawn from a previous international Delphi consensus process [21]. Nevertheless, inclusion of a microbiologist, ward nurse and specialist team representatives may have further contributed to our findings [16]. Both focus groups were recorded and transcribed verbatim providing confirmation of the results, with participant validation. Finally, we drew on the Behaviour Change Wheel [24], to develop the intervention package and to formulate the mechanisms of action through which the intervention components are expected to impact key outcomes. However, it should be noted that as the focus groups represented UK clinical practice, the findings are limited to similar universal healthcare systems with comparable system and staff resources.

The intervention package is designed to support ICU systems and prioritise staff engagement and, in doing so, address many of the barriers to intervention delivery identified. The intervention package now needs to be tested in clinical practice to confirm deliverability and clinical acceptance within different clinical contexts. Some of the intervention components, e.g., post-transfer medicines reconciliation, may be already implemented into practice in ICUs variably [41, 42], but experience some of the barriers we have identified [43] Acknowledging the key barriers to complex intervention delivery is crucial to improving patient care on transfer from ICU [42], and demonstrating the feasibility of routine delivery in practice prior to routine adoption and implementation [19].

## Conclusions

We developed a theory-informed intervention package to address the limitations in medication safety for ICU patients transferring to a hospital ward. Understanding what needs to change, complimented by the accompanying facilitators, will aid implementation and refinement of the complex intervention package prior to wider evaluation in a future multicentre study.

## Supplementary Information

Supplementary Material 1.

## Data Availability

The datasets used and/or analysed during the current study are available from the corresponding author on reasonable request.

## Abbreviations

ACCP: Advanced Critical Care Practitioner
ICU: Intensive Care Unit
UK: United Kingdom
