# Supplementary material for: Improving medication safety for intensive care patients transitioning to a hospital ward: development of a theory-informed intervention package
Source: BMC Health Serv Res. 2024 Nov 26;24:1476. doi: 10.1186/s12913-024-11627-3 (PMC11600792; doi:10.1186/s12913-024-11627-3)
Supplement: Supplementary file 1 — Supplementary Material 1. [file 12913_2024_11627_MOESM1_ESM.docx]

## Topic Guide (1^st^ Focus Group)

**Part 1: Identification of what needs to change (intervention targets)**

What do we need to change to improve medication safety for ICU patient transitions?

What do we need to target?

**Part 2: Identification of intervention “Core” components**

(a) Explore importance and reasoning

Which intervention components do you think are the most critical (core) for these intervention targets?

**Task 1. [1-2-4-All Exercise]**

What are your reasons for these views?

**Task 2. [Value and complexity matrix]**

(b) Prioritising, targeting and delivery

For each intervention component post-it notes applied to indicate assigned prioritisation, targeting and delivery

What level are the intervention components targetted (e.g. individual, team, organisation)?

What behaviour change techniques are needed (e.g. providing information, skill development)?

How should these be delivered (e.g. training sessions, guidelines, checklists)?

Can these core intervention components be delivered during periods of time constraints e.g. out of hours? If not, what alternative systems could be used e.g. better discharge planning?

How can we bridge “work as done” and “work as imagined”?

If patient and family engagement is important, how could we deliver increased engagement?

**Part 3:** **What will the intervention change (mechanism of action) and what are the desired outcomes**

Have post-it notes available with all the important patient and medication outcomes for the eDelphi

What will change as a result of the interventions (mechanisms of action)?

How do these link to the important medication and patient outcomes?

**Part 4: Understanding the barriers and facilitators to intervention delivery**

**Task 3: “insurmountable barriers” and “achievable facilitators”**

Thinking of the **core** intervention components you have identified, what are the key barriers and facilitators to their delivery?

How do we optimise the key facilitators?

How should we overcome these key barriers within existing resources?

Where is there routine duplication of work or potential redundancy in the process?

## Focus Group Tasks

### Part 2: Identification of intervention “Core” components (50mins)

What do we want to answer?

- Which intervention components do you think are the most critical?
- What are your reasons for these views?

**Task 1. 1-2-4-All** (25mins)

Task instructions

- On your own, spend **5 mins** looking at the important interventions that have been identified through the Delphi/HTA etc, identify which ones you think **are the most critical for the intervention targets identified** (there is no right nor wrong!). Stick those that you think form your “core intervention components” on the laminated A3 sheet. (Facilitators remove all those that have not been identified as key at this stage.
- Move into your pair and spend **5 mins** working together to identify which of the core intervention components you agree on, and try to arrive at a consensus on those that you do not
- Move into your group of 4 and repeat the above task **(5 mins)**
- Return to the full group and repeat the above **(10 mins)**

Facilitator prompts

- Why do you think these are the core intervention components? (Reasoning)

### Task 2. Value and complexity matrix (15mins)

Task instructions

- As a group we would like for you to use the value and complexity matrix and identify which of the key components you believe fit into which part of the circle

Facilitator prompts

- Why do you think they have lower/higher clinical importance?
- What makes that intervention harder to implement consistently?

### Task 3. Understanding barriers and facilitators to intervention delivery (10mins)

What do we want to answer?

- Thinking of the **core** intervention components you have identified, what are the key barriers and facilitators to their delivery?
- How do we optimise the key facilitators?
- How should we address these key barriers?
- Are these acheivable with existing resources? If not, are there other ways to address?
- Where is there routine duplication of work or potential redundancy in the process?

Task instruction

- Along the ribbon discuss as a group and decide where each of the packaged core intervention components sit on a scale between- “insurmountable barriers” and “achievable facilitators”

Facilitator instructions

- How do we optimise key facilitators
- How can we address key barriers
- Are these achievable with existing resources?

## Topic Guide (2nd Focus Group)

**Part 1: Confirmation of what needs to change (intervention targets)**

What do we need to change to improve medication safety for ICU patient transitions?

What do we need to target?

Show Results from 1^st^ Focus Group

*Do you agree?*

*Anything we have missed?*

**Part 2: Confirmation of intervention “Core” components**

Which intervention components do you think are the most critical (core) for these intervention targets?

Show Results from 1^st^ Focus Group

*Do you agree?*

*Anything we have missed? Specifically ask re: roles of individual & team referrals*

What are your reasons for these views?

**Task 1. [Value and complexity matrix]**

Update for any new additions of “core” intervention components **by adding to matrix**

1. Prioritising, targeting and delivery

Show Results from 1st Focus Group

For **any** **NEW** intervention component use flip chart to record prioritisation, targeting and delivery

What level are the intervention components targetted (e.g. individual, team, organisation)?

What behaviour change techniques are needed (e.g. providing information, skill development)?

How should these be delivered (e.g. training sessions, guidelines, checklists)?

1. Packaging at “mode of delivery”

Using all modes of delivery identified, develop the objectives & learning outcomes.

For each Intervention Component - ask "Who does it?", "When they do it?" and "How they do it"?

Can these core intervention components be delivered during periods of time constraints e.g. out of hours? If not, what alternative systems could be used e.g. better discharge planning?

How can we bridge “work as done” and “work as imagined”?

Where is there routine duplication of work or potential redundancy in the process?

If patient and family engagement is important, how could we deliver increased engagement?

1. Packaging of intervention components

Show Results from 1st Focus Group

Do you agree?

Would you change anything?

**Part 3: What will the intervention change (mechanism of action) and what are the desired outcomes**

Show Results from 1st Focus Group

What will change as a result of the interventions (mechanisms of action)?

How do these link to the important medication and patient outcomes?

(Note: it may be that medication-related outcomes are the MoA leading to the patient outcomes) *e.g. Medication reconciliation in ICU admisison -> reduces medciation errors -> reduces patient adverse (drug) events*

**Part 4: Understanding the barriers and facilitators to intervention delivery**

**Task 2: “insurmountable barriers” and “achievable facilitators”**

Show Results from 1st Focus Group

*Do you agree?*

*Any you would add or remove?*

*Thinking of the* ***core*** *intervention components you have identified, what are the key barriers and facilitators to their delivery?*

How do we optimise the key facilitators?

How should we overcome these key barriers within existing resources?


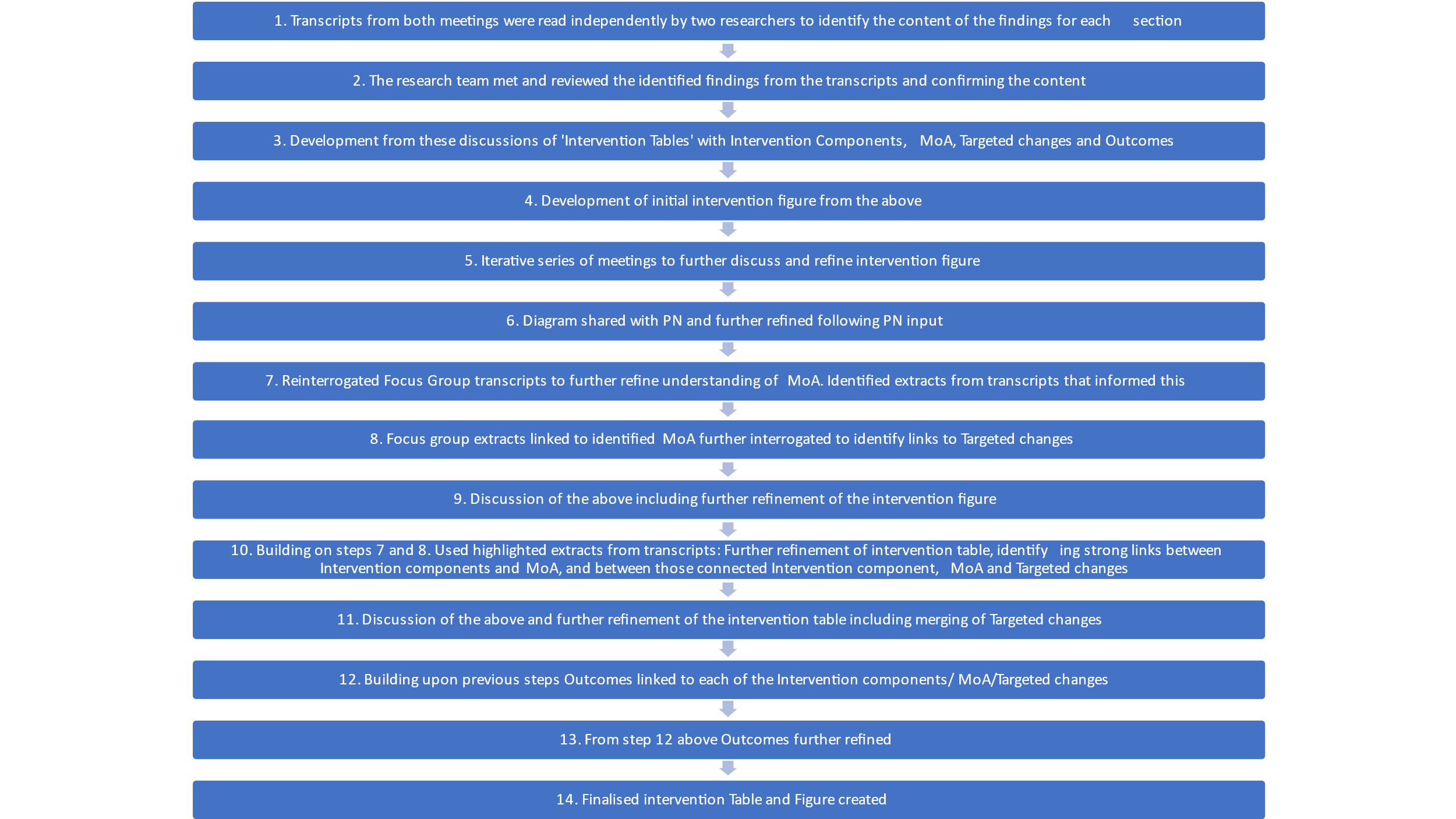


**Figure A1:** **Iterative process for confirming the relationship between the intervention components, mechanism of action, targeted changes and outcomes**

MoA: Mechanism of Action; PN: Professor Paul Norman

| PHASE 1 - IDENTIFY LINKS BETWEEN INTERVENTION COMPONENT/MoA |
| --- |
| **Strong links defined by:** |
| 1. Multiple times (3 or more) link has been highlighted for specific Intervention component |
| 2. Coherence and clarity of the narrative - in highlighted extract. |
| Strength of links in order |
| 1 & 3 Multiple times (3 or more) MoA link has been highlighted for specific Intervention component AND Coherence and clarity of the narrative |
| Multiple times (3 or more) MoA link has been highlighted for specific Intervention component |
| Coherence and clarity of the narrative |
| PHASE 2 - IDENTIFY STRONG TARGETED CHANGES WITHIN RESULTS FROM PHASE 1 (SO ONLY WHERE STRONG INTERVENTION COMPONENT/MoA LINK ALREADY IDENTIFIED) |
| 1. Occur two or more times per Intervention Component/MoA link |
| 2. Occur two or more times per Intervention component across the MoAs that the Intervention is connected to (highlighted at each Intervention/MoA link for clarity) |
| 3. Both 1 and 2 |

**Table A1: Identify relationships between Intervention components, Mechanisms of Action, Targeted Changes and Outcomes**

MoA: Mechanism of Action


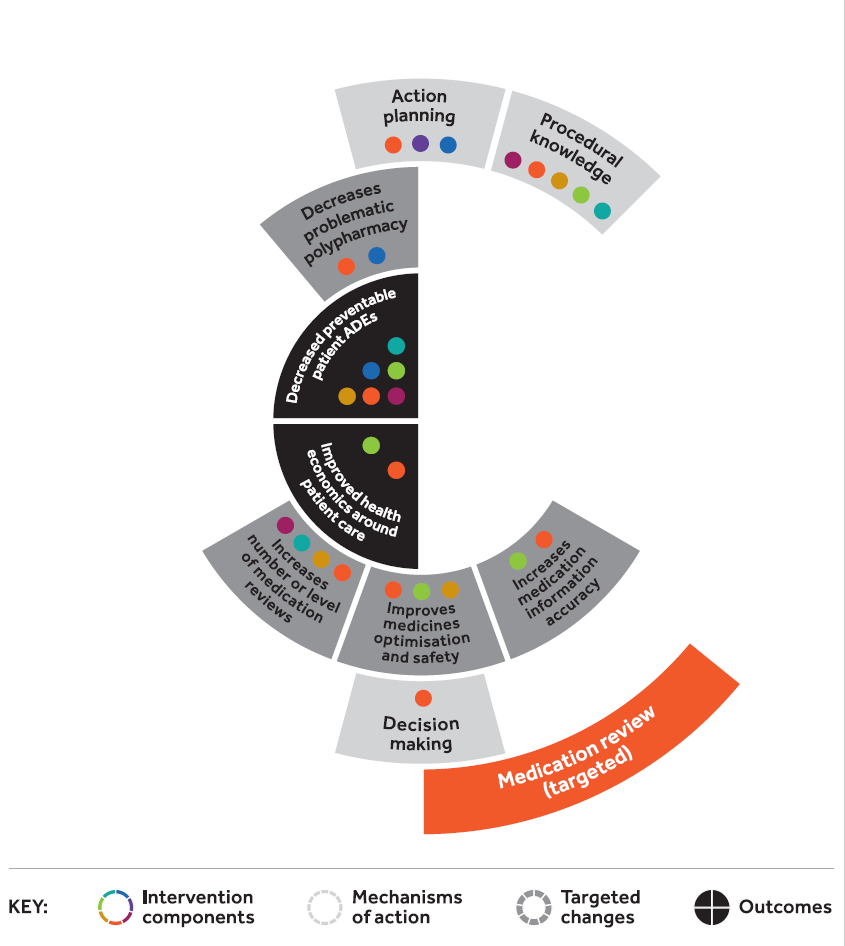


**Figure A2. Medication review intervention relationships**

**
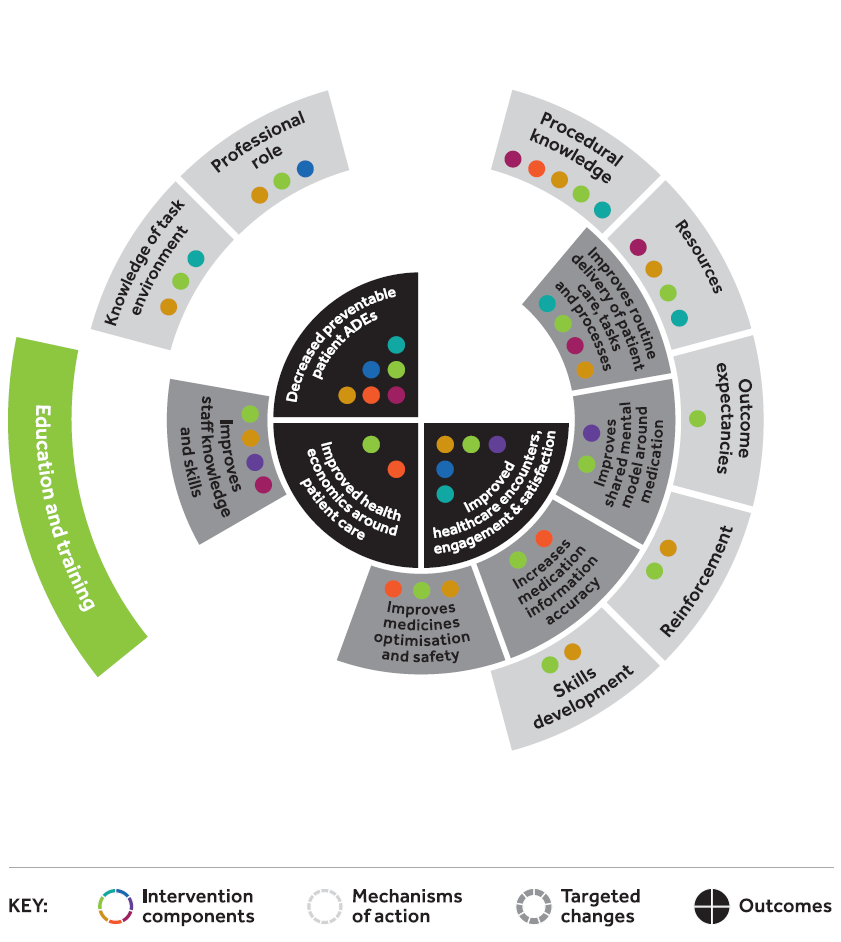
**

**Figure A3. Education and Training intervention relationships**

**
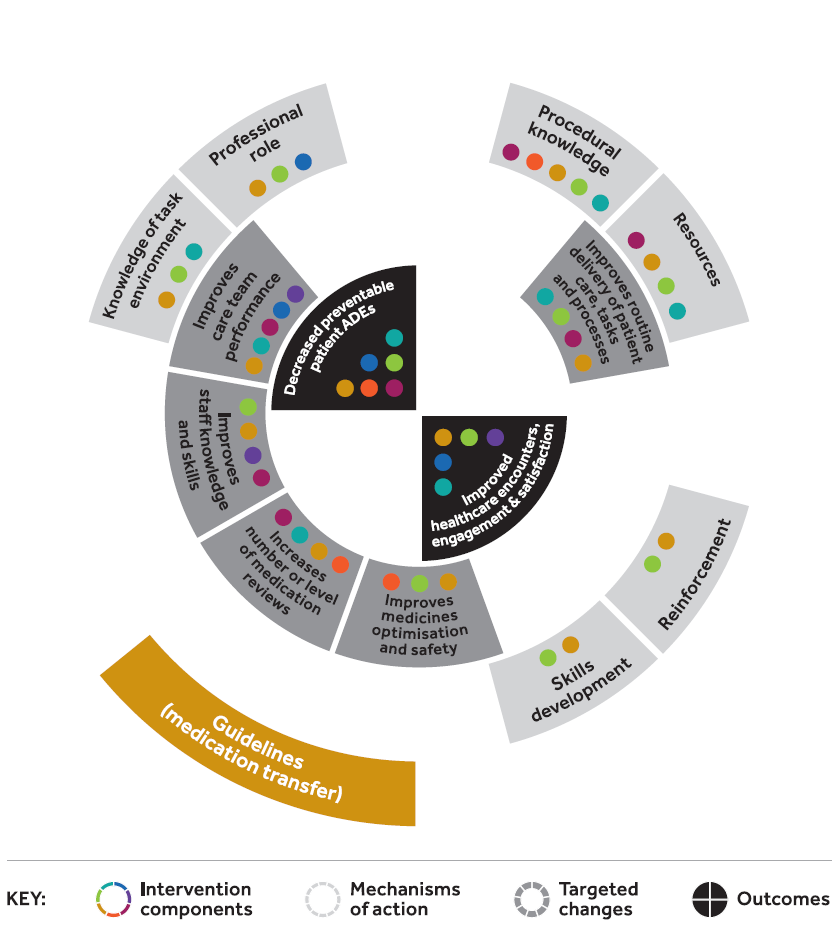
**

**Figure A4. Guidelines interventions relationships**

**
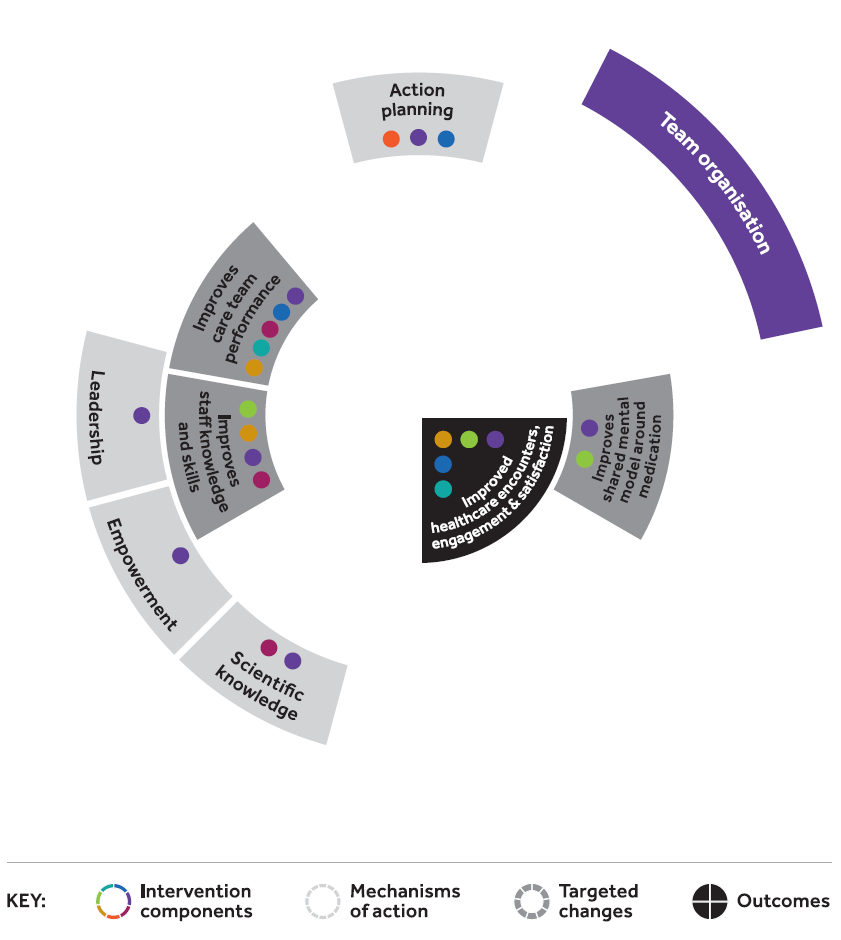
**

**Figure A5. Team Organisation intervention relationships**

**
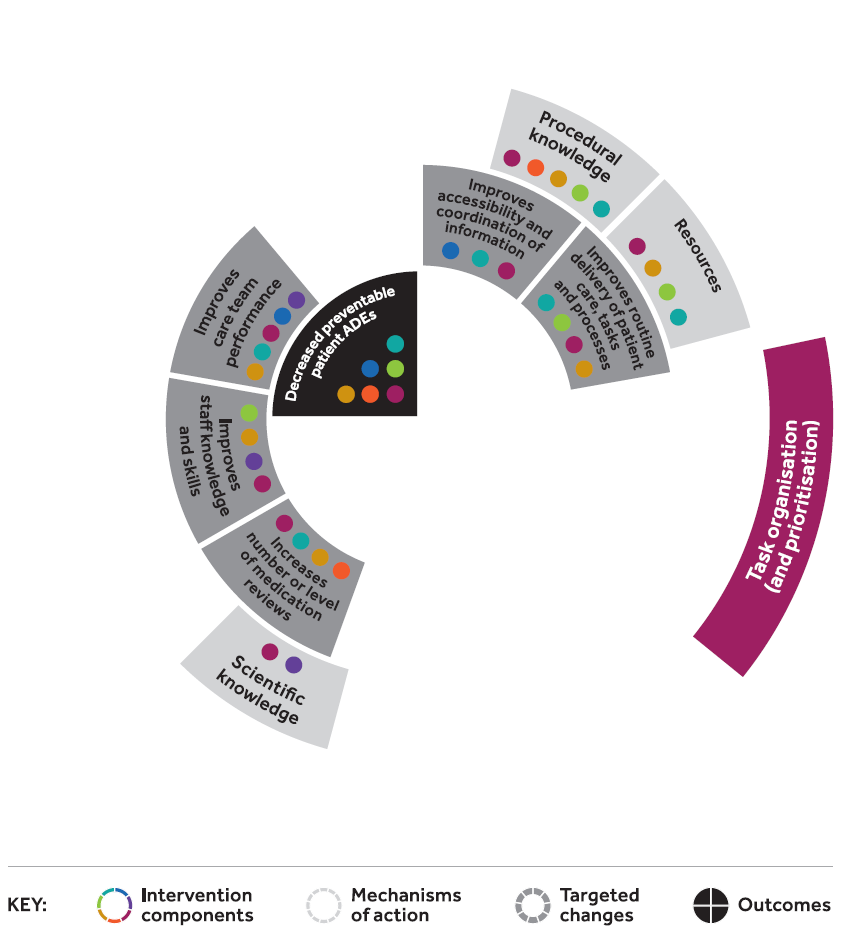
**

**Figure A6. Task Organisation intervention relationships**

**
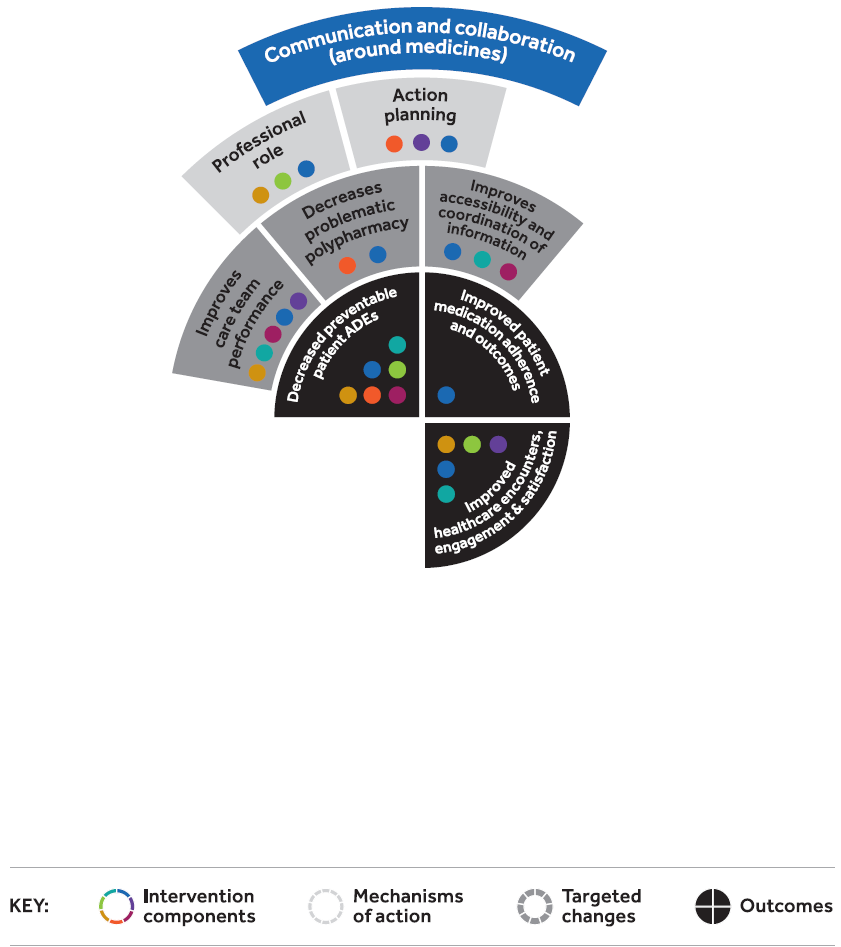
**

**Figure A7. Communication and Collaboration intervention relationship**

**
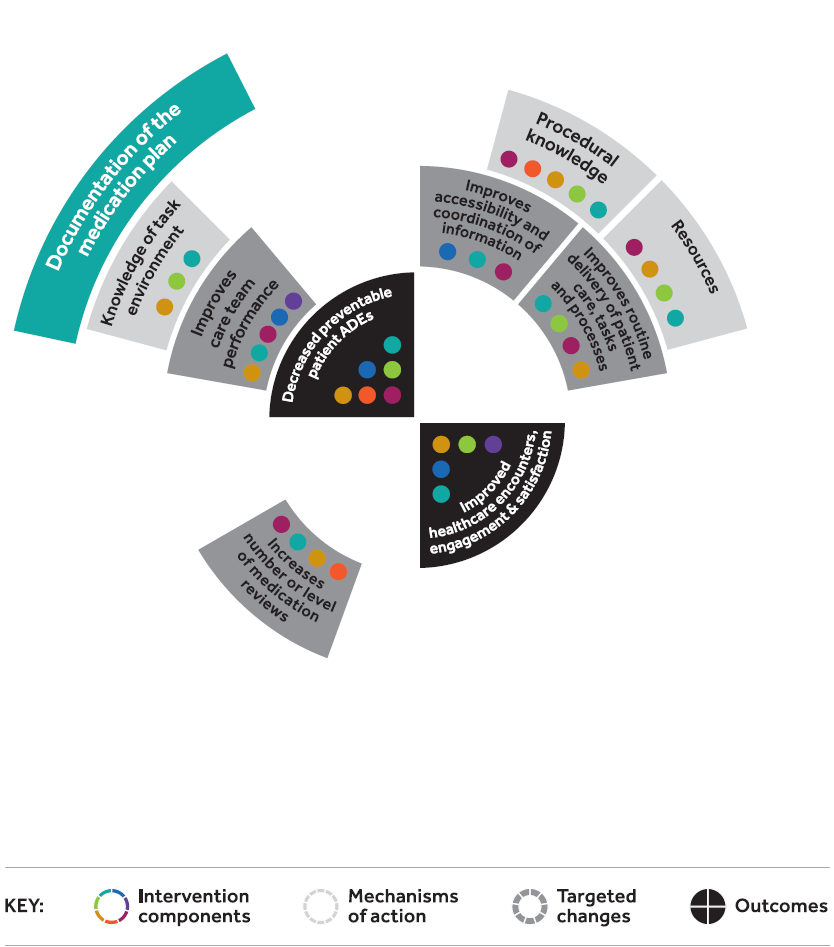
**

**Figure A8. Documentation of the medication plan intervention relationships**
